# Recycling of Plastics from E-Waste via Photodegradation in a Low-Pressure Reactor: The Case of Decabromodiphenyl Ether Dispersed in Poly(acrylonitrile-butadiene-styrene) and Poly(carbonate)

**DOI:** 10.3390/molecules28062491

**Published:** 2023-03-08

**Authors:** Hussam Aldoori, Zohra Bouberka, Hervé Feuchter, Skander Khelifi, Franck Poutch, Loic Brison, Fouad Laoutid, Stijn Steuperaert, Corinne Foissac, Philippe Supiot, Christian Malas, Ulrich Maschke

**Affiliations:** 1 Unité Matériaux et Transformations-UMET, UMR 8207, University of Lille, CNRS, INRAE, Centrale Lille, 59000 Lille, France; 2 Laboratoire Physico-Chimie des Matériaux-Catalyse et Environnement (LPCMCE), Université des Sciences et de la Technologie d’Oran-Mohamed Boudiaf (USTOMB), Oran 31000, Algeria; 3 CREPIM, Rue Christophe Colomb, Parc de la Porte Nord, 62700 Bruay-la-Buissière, France; 4 Materia Nova Research Center, Laboratory of Polymeric and Composite Materials (SMPC), 7000 Mons, Belgium; 5 Centexbel, Technologiepark 70, 9052 Gent-Zwijnaarde, Belgium; 6 Institut Chevreul, Cité Scientifique, Avenue Paul Langevin, 59655 Villeneuve d’Ascq, France

**Keywords:** recycling, waste from electrical and electronic equipment, brominated flame retardant, decabromodiphenylether, Poly(acrylonitrile-butadiene-styrene), Poly(carbonate), photodegradation

## Abstract

Recycling of plastic waste from electrical and electronic equipment (EEE), containing brominated flame retardants (BFR) remains difficult due to the increasingly stringent regulations on their handling and recovery. This report deals with photodegradation in a low-pressure reactor applying UV-visible light on Decabromodiphenyl ether (DBDE or BDE-209) randomly dispersed in commercially available Poly(acrylonitrile-butadiene-styrene) (ABS) and Poly(carbonate) (PC). The aim of this study is to investigate the possibility of decomposing a BFR in plastic waste from EEE while maintaining the specifications of the polymeric materials in order to allow for their recycling. The photodegradation of the extracted BFR was monitored using infrared spectroscopy and gas chromatography coupled with mass spectroscopy. DBDE underwent rapid photodegradation during the first minutes of exposure to UV-visible light and reached degradation yields superior to 90% after 15 min of irradiation. The evaluation of polymer properties (ABS and PC) after irradiation revealed superficial crosslinking effects, which were slightly accelerated in the presence of DBDE. However, the use of a low-pressure reactor avoids large photooxidation and allowed to maintain the thermal and structural properties of the virgin polymers.

## 1. Introduction

The flagship waste treatment directive (2012/19/EU) requires the selective treatment of plastics containing Brominated Flame Retardants (BFRs, 10–30% by weight), which are present in Waste Electrical and Electronic Equipment (WEEE) [1,2,3]. At the European level, the amount of WEEE is growing at a rate of more than 2% per year (for example, the total volume of this waste currently represents more than 115kt in France and Belgium). This waste contains 17–25% of plastic materials of which at least 36–40% by weight are additivated with BFRs.

**Figure 1:**
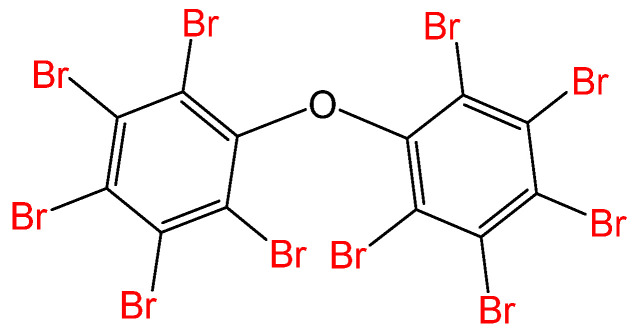
Structure of decabromodiphenylether (DBDE).

PBDEs (polybrominated diphenyl ethers) and PBBs (polybrominated biphenyls) are among the molecules that were widely used in the past for the flame retardant treatment of plastics [1,2,3]. Decabromodiphenylether (DBDE) (Figure 1) was one of the most applied BFRs in plastics from EEE, toys, textile, and furniture to meet fire safety regulations [1,2]. It has been phased out in a lot of countries in Europe [3], North America [4], and Asia [5] due to its bioaccumulation and toxicity [6,7,8]. In fact, due to its persistent properties, it has become an ubiquitous pollutant in the environment [9,10]. However, DBDE is still found in waste from obsolete EEE (WEEE) [11], and is estimated to remain for years to come in these materials [12]. In fact, E-waste handling facilities are recognized as an important source of release of BFRs into the surrounding environment [13,14,15,16]. Moreover, reckless recycling practices have indeed unintentionally put BFRs in articles that are not required to meet flame retardancy requirements [17,18]. Currently, European regulations restrict recycling of all materials containing polybromodiphenyl ethers (PBDEs), and advises the destruction of these molecules [19]. High temperature incinerators are used as a solution for energy recovery of these materials [20]. However, this process requires a throughout gas cleaning as a thermal treatment for DBDE, representing a source of production of highly toxic polybrominated dibenzofurans (PBDF) and dibenzodioxins (PBDD) [21,22]. Furthermore, DBDE can degrade in the environment to lower brominated diphenyl ethers [23,24], which are regarded as having a greater bioaccumulation potential [7,25], mainly by photo-induced degradation. PBDEs are now considered as widespread environmental pollutants, raising health concerns, which have encouraged researchers to find ways for their remediation [26]. The photodegradation behavior of PBDEs has been widely investigated in various media: in air [27], in organic solvents [24,28,29,30], in aquatic media [31], and much less intensively in solid phase [24,32,33]. Khaled et al. reported on the effect of the solid matrix on the degradation kinetics of BFRs in plastics [34]. Moreover, photoremediation has been considered as a promising technique of decontamination of persistent organic pollutants [35]. However, in the case of plastic decontamination, the exposure to UV-light can deteriorate considerably the materials by photo-oxidation effects [36,37]. The oxidation of the polymer surface was found to slow down the photodegradation of BFRs [34]. It is thus important to limit the occurrence of these oxidation effects.

In this study, photodegradation of DBDE dispersed in polymer matrices, induced by UV-visible light, was carried out in a low-pressure reactor. The latter allowed to reduce substantially the presence of oxygen during light exposure. Poly(acrylonitrile-butadiene-styrene) (ABS) and Poly(carbonate) (PC) were chosen since they were often flame retarded with DBDE, and represent thus large fractions in WEEE [38,39]. ABS and PC are also commonly blended for different applications in EEE [40]. Since polymers themselves are generally vulnerable to UV-light even in oxygen-free atmospheres [41], an evaluation of the properties of irradiated polymers under reduced pressure is necessary to assess the feasibility of such decontamination processes of plastic waste, allowing to proceed to their valorization.

Photodegradation studies of BFR in plastics are scarce, and to our knowledge this is the first report that investigates both the abatement effect of BFR and the assessment of decontaminated polymers, applying UV-visible irradiation under reduced pressure.

## 2. Materials and Methods

### 2.1. Chemicals 

Commercial DBDE (GC Deca 83) was purchased from Greenchemical S.p.a. (Desio MB, Italy), and its chemical structure is given in Figure 1. ABS Polylac^®®^ was purchased from Chi Mei Corporation (Tainan, Taïwan) through AMP Polymix (Horbourg-Wihr, France) and PC Makrolon^®®^ was obtained from Covestro AG (Leverkusen, Germany). Tetrahydrofuran (THF) and Toluene (HPLC grade solvents) were purchased from Sigma Aldrich (Steinheim, Germany) and Petroleum Ether from VWR International GmbH (Darmstadt, Germany). Tetrabromobisphenol A (TBBPA), used as an external standard, was purchased from Sigma Aldrich (Steinheim, Germany).

### 2.2. Sample Preparation

Model polymer/BFR composites were prepared containing 10 wt.% DBDE, that is within the usual range of the BFR concentrations in plastics from WEEE. To elaborate these blends, a twin-screw extruder DSE-25 from Brabender GmbH and Co. KG (Duisburg, Germany) was used at 210 °C for the ABS-based composite and at 250 °C for the PC blends. Thin films of 60–100 µm were produced using a laboratory molding press (Servitec Polystat 200T) (Servitec Maschinenservice GmbH, Wustermark, Germany) applying 90 bars for 2 min at 180 °C for ABS and at 230 °C for PC.

Blends based on ABS and PC containing 90 wt-% virgin polymer + 10 wt-% of the irradiated blend (90 wt-% polymer/10 wt-% DBDE) were prepared using a MiniLab II Haake Micro Compounder from Thermo Fisher Scientific (Illkirch-Graffenstaden, France) at 240 °C (3 min at 30 rpm followed by 5 min at 75 rpm).

A mini-injection machine (Haake Minijet Pro from Thermo Scientific, Dreieich, Germany) was applied to prepare specimens for tensile testing (ASTM D638 type V). The injection molding parameters were set as follows: temperatures of the barrel 240 °C for ABS and 250 °C for PC; mold at 45 °C; time for preheating and melting 90 s; followed by a period of 10 s at 300 bars and 5 s at 200 bars.

### 2.3. Irradiation Experiments

Irradiation of ABS and PC containing 10 wt.% DBDE was carried out at an ambient temperature using a LC8 Xenon light source from Hamamatsu Photonics France S.A.R.L. (Massy, France), equipped with an optical fiber. This light source covers a broad spectrum from UV- to visible-light similar to the sunlight spectrum. Air-free irradiation experiments were conducted in a home-made vacuum-sealed polytetrafluoroethylene cell equipped with a quartz window on the top, allowing to fix the optical fiber. The cell was connected to a primary vacuum pump to keep the pressure below 20 mbars. The polymeric films were mounted in the cell at an angle of 45° to allow for simultaneous irradiation and an FTIR analysis. The optical fiber was placed vertically while the IR bundle went horizontally through the films.

### 2.4. Ultrasonic-Assisted Extraction and Purification

The irradiated polymer films were submitted to a solvent-based method to extract the dissolvable species (the remaining DBDE, photoproducts, such as lower brominated species, and other additives, etc.) for analysis purposes, applying the following procedure: 20 mg of polymer was swelled in 2 mL toluene in an ultrasound bath (Elma Schmidbauer GmbH, Singen, Germany) for 30 min at 70 °C. The polymer was then precipitated by adding petroleum ether, and the surfactant was centrifuged at 4000 rpm. The recovered solution of petroleum ether/toluene containing the photoproducts, additives, and traces of polymer was further purified using a silica column (diameter: 2 cm; length: 20 cm) and eluted with petroleum ether. The solvent was evaporated from the recovered solution and the solid extract was dried overnight under vacuum at 60 °C. The extraction efficiency was monitored by Fourier-transform infrared spectroscopy (FTIR) and an X-ray fluorescence spectrometry (XRF) analysis, indicating a recovery superior to 96% and 98% for the PC and ABS mixtures, respectively.

### 2.5. Instrumentation

The FTIR spectroscopy analysis was carried out on thin films using a Frontier spectrometer from Perkin Elmer (Perkin Elmer, Waltham MA, USA). The in situ analysis in the transmission mode was executed in order to analyze in depth changes, using a cell mounted directly in the IR trajectory of the spectrometer. The surface analysis of the films was carried out using the attenuated total refraction (ATR) accessory.

The gas chromatography–mass spectrometry (GC/MS) analysis was performed on a Perkin Elmer Clarus 680 gas chromatograph using an Elite-XLB capillary column of 30 m × 0.25 mm with a film thickness of 0.10 μm, coupled with a Clarus 600T mass detector (PerkinElmer, Waltham, MA, USA) equipped with a quadrupole mass analyzer (QSM). The injector temperature was set at 325 °C. Helium was the carrier gas, with a constant flow of 1.5 mL min^−1^. Electron ionization (EI) was used with an ion source temperature of 180 °C and an interface temperature of 300 °C, with the EI spectra obtained at 70 eV. A *m*/*z* range between 40 and 1100 was scanned. The initial program temperature was set at 120 °C, followed by a ramp of 10 °C min^−1^ to 300 °C and an isotherm of 30 min. Tetrabromobisphenol A was used as an external standard for the quantification at a concentration of 50 ppm, and every experiment was repeated several times to insure an acceptable reliability. 

The quantitative measurements of the remaining lower brominated PBDE concentrations were conducted by Atmospheric Pressure Photo Ionization-Liquid Chromatography coupled with a Tandem mass spectrometer (APPI-LC-MS/MS). An Agilent 1290 UPLC system (Agilent, Santa Clara, CA, USA) was used with an ACE3 C18 column of 50 mm × 2.1 mm coupled with a Sciex QTRAP^®®^ 4500 triple quadrupole system with an APPI source. The injection volume was 5 µL and the column was maintained at 20 °C. A gradient of water/methanol was used at a flow rate of 0.3 mL min^−1^. A dopant consisting of an acetone/toluene mixture was injected at a rate of 60 µL min^−1^ to enhance the ionization in the APPI source.

The total bromine content was determined using a Hitachi SU8020 Scanning Electron Microscope (SEM) (Hitachi Ltd., Tokyo, Japan), coupled to an Energy Dispersive X-ray Spectrometer analyzer (EDX).

The glass transition temperatures of the polymers before and after irradiation were measured by means of Differential Scanning Calorimetry (DSC 8000 instrument, PerkinElmer, Waltham, MA, USA). The samples were prepared by introducing 8–12 mg of the polymers into aluminum pans. Three successive heating and cooling ramps of 10 °C min^−1^ were applied in the temperature range from −50 °C to 200 °C under nitrogen flow.

The gel permeation chromatography (GPC) measurements were performed at room temperature on a Waters Alliance e2695 system (Waters S.A.S., Saint-Quentin en Yvelines, France) using THF as a solvent (flow rate 1 mL min^−1^) equipped with three columns placed in series (Styragel HR1, Styragel HR3, and Styragel HR4) and coupled to two detectors: A Wyatt RI (Differential Refractive Index) detector and a Wyatt MALS (Multi Angle Light Scattering) detector (laser wavelength: 670 nm). The calibration was established with polystyrene standards. Each time, a fixed amount of 5 mg of the sample was dissolved in 5 mL of THF for 30 min in an ultra-sonic bath. The solution was then centrifuged at 4000 rpm and filtered using 0.20 µm filters to remove any undissolved particles.

The tensile testing measurements were performed using a Lloyd LR 10K tensile bench (TTS Ltd., Worthing, West Sussex, UK) at a speed rate of 10 mm min^−1^ using a distance of 25.4 mm between grips. An analysis was carried out on the specimens that were previously conditioned for 48 h at 20 ± 2 °C and a relative humidity of 50 ± 3%; the values were averaged over six measurements.

## 3. Results and Discussion

### 3.1. Photodegradation Efficiency

#### 3.1.1. Fourier-Transform Infrared Spectroscopy

**Figure 2:**
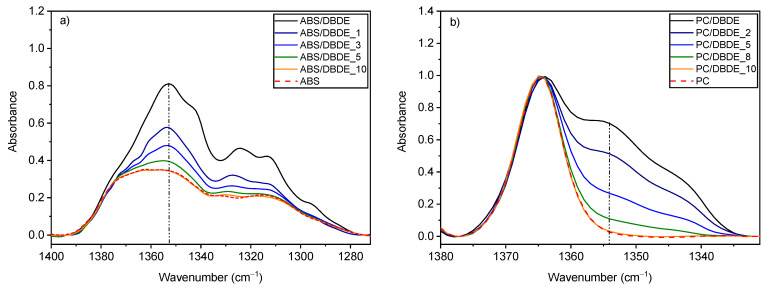
FTIR spectra of (**a**) 90 wt-% ABS/10 wt-% DBDE and (**b**) 90 wt-% PC/10 wt-% DBDE films: Evolution of the ether band of DBDE at 1350 cm^−1^ as function of irradiation time given in minutes.

The FTIR spectra of 90 wt-% ABS/10 wt-% DBDE (Figure 2a) and 90 wt-% PC/10 wt-% DBDE (Figure 2b) films reveal a characteristic band of DBDE with a peak situated between 1350 cm^−1^ and 1354 cm^−1^, assigned to the C-O-C ether band of DBDE. This band was followed up to determine the effect of the photodegradation on the target molecule (DBDE). Irradiation experiments demonstrated a rapid decrease in the C-O-C band in both polymer/BFR films during the first 10 min of irradiation as shown in Figure 2, of which the first 3 min experienced a high reaction rate.

**Figure 3:**
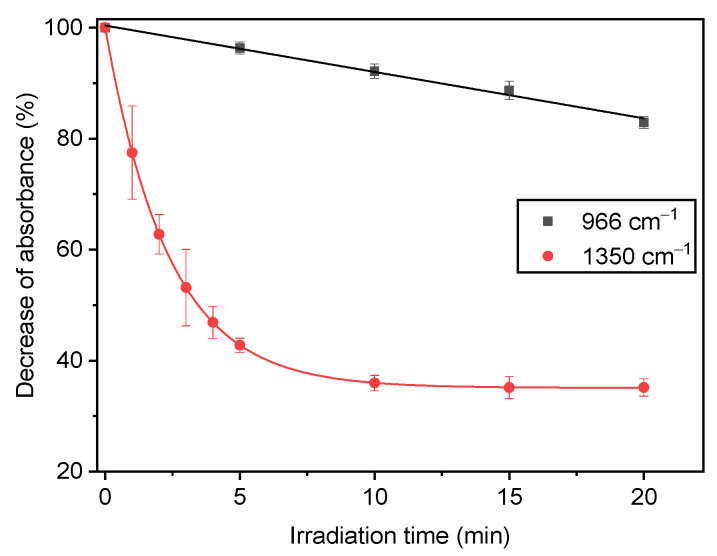
Absorbance decline of two FTIR bands in 90 wt-% ABS/10 wt-% DBDE film, relative to their initial values.

The bands at 966 cm^−1^ and 911 cm^−1^, attributed to the vinyl stretching of the butadiene component, can be regarded as the most fragile part of the ABS chain [42]. The band at 966 cm^−1^ showed a rather linear decrease as a function of irradiation time; however, this was small when compared to the decrease in the 1350 cm^−1^ band, which stabilized after a certain irradiation time as can be observed in Figure 3. It should be mentioned that the absorbance at 1350 cm^−1^ was not expected to fall below a certain value in the irradiated ABS/DBDE blends since the virgin ABS presents a specific absorbance around this wavenumber as can be seen in Figure 2a. In the case of the PC (Figure 2b), the disappearance of the shoulder at 1350 cm^−1^ was rather complete after 10 min of exposure time with no visible change in the other bands.

#### 3.1.2. Gas Chromatography–Mass Spectrometry

**Figure 4:**
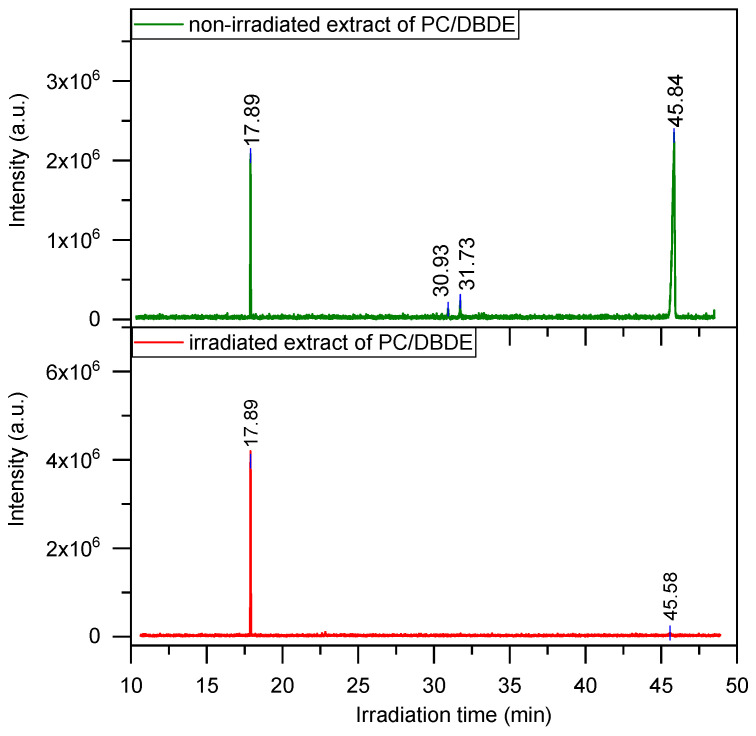
GC chromatograms of extracted solutions from 90 wt-% PC/10 wt-% DBDE films before and after irradiation for 10 min under reduced pressure.

**Figure 5:**
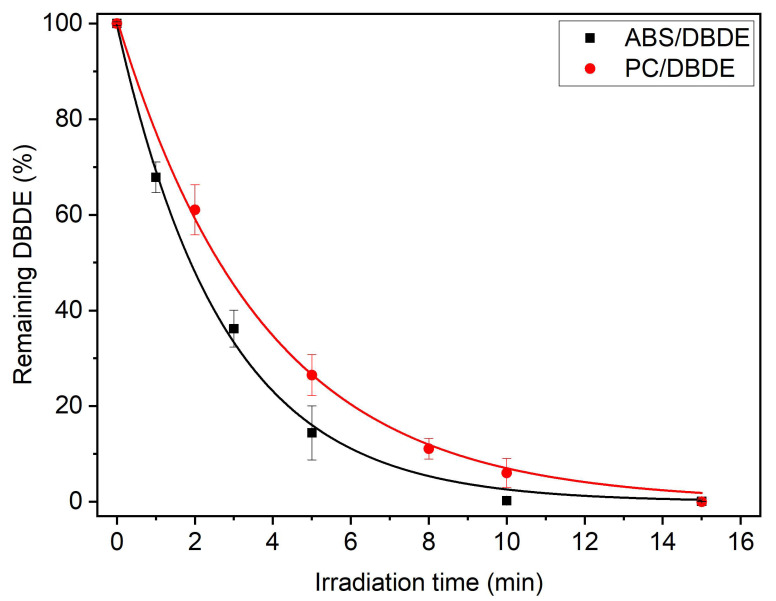
Decay profiles of remaining DBDE from 90 wt-% ABS/10 wt-% DBDE and 90 wt-% PC/10 wt-% DBDE films, obtained by GC-MS.

The kinetics of photodegradation of DBDE was followed by GC/MS after ultrasound assisted extraction and purification of the extracts. The extracts from the non-irradiated (90 wt-% ABS/10 wt-% DBDE) and (90 wt-% PC/10 wt-% DBDE) contain predominantly DBDE and traces of different isomers of Nona-BDE (impurities present in the commercial DBDE), which mostly disappeared after irradiation as can be observed in the chromatograms presented in Figure 4. The fixed concentration of the external standard allowed to quantify the amount of the remaining DBDE using a calibration curve. The photodegradation profiles of DBDE, presented in Figure 5, exhibit exponential decays obeying the pseudo-first order reaction rate. The reaction generates lower brominated congeners, in accordance with the widely reported stepwise debromination mechanism, following the cleavage of the C-Br bond [32,34]. The photodegradation reaction rates of DBDE, averaged over at least four experiments, were slightly higher in ABS than in PC. Indeed, the rate constants were 0.36 min^−1^ and 0.27 min^−1^ for ABS and PC, respectively.

The trace analysis, carried out using the APPI-LC/MS-MS technique, revealed a remaining concentration of DBDE of 0.89% and 1.58% in ABS and in PC, respectively. Hence, the conversion yield of photodegradation exceeds 85% in all the conducted experiments, which represents a significant increase from the previous experiments conducted in the presence of oxygen from the atmosphere [43,44]. Furthermore, the enhanced stability of ABS and PC to UV-visible light exposure, under reduced pressure, allowed for the application of increasing the radiation dose before oxidation effects took place.

The mass balance imposes conservation of the overall bromine content before and after irradiation. However, the mass of all the detected brominated photoproducts in both the polymer/BFR systems was estimated to be lower than that of the initial DBDE, suggesting that volatile organic compounds (VOC) were released, containing bromine compounds.

Bromine content measurements of the films by the EDX analysis confirmed a decrease situated between 88% and 97% of the total bromine content on both the film surfaces after 15 min irradiation time, which was in good agreement with the conversion yields of DBDE mentioned above.

### 3.2. Polymer Properties after UV-Visible Light Exposure

#### 3.2.1. ATR-FTIR Analysis

**Figure 6:**
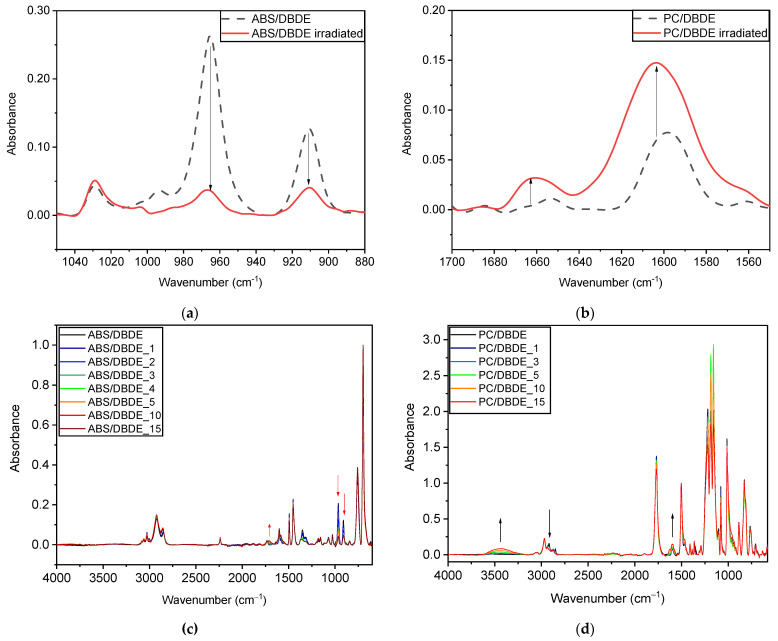
Evidence of surface alteration of irradiated samples in ATR-FTIR spectra (**a**) Decrease in butadiene bands (90 wt-% ABS/10 wt-% DBDE film); (**b**) Emergence of hydroxybenzophenone bands (90 wt-% PC/10 wt-% DBDE film); (**c**) Evolution of the full spectrum of 90 wt-% ABS/10 wt-% DBDE film with irradiation time given in minutes; (**d**) Evolution of the full spectrum of 90 wt-% PC/10 wt-% DBDE film as function of irradiation time given in minutes.

The evolution of the FTIR bands, attributed to the polymer parts, as a function of the irradiation time was examined by the ATR analysis on both sides of the films. Figure 6a,c show the result from the light-exposed surface of the 90 wt-% ABS/10 wt-% DBDE film, whereas Figure 6b,d present the data of the 90 wt-% PC/10 wt-% DBDE film. A decrease in bands at 966 cm^−1^ and 911 cm^−1^ was observed, which might correspond to the evolution of the unsaturated vinyl groups from the butadiene part of ABS (Figure 6a). A broadening of the carbonyl stretching band around 1730 cm^−1^ was also recognized. A stretching band at 3500 cm^−1^ (O-H bond) for the 90 wt-% PC/10 wt-% DBDE film appeared only at the more extended light exposure times (Figure 6d). The emergence of weak bands at 1600 cm^−1^ and 1660 cm^−1^ was observed only on the irradiated side of this film (Figure 6b), indicating the possibility of photo-Fries rearrangement as a result of UV-radiation [45]. A decrease in the C-H stretching bands at 2920 cm^−1^ and 2850 cm^−1^ also indicates the eventual crosslinking effects at the exposed surface of PC-containing films.

The changes appearing to ABS and PC due to the UV-light irradiation seem to take place on the exposed surfaces rather than occurring in the bulk. Additionally, very little oxidation effects were detected during the irradiation under the reduced pressure for both polymers, increasing thus their stability when compared to open air irradiation [43,44].

#### 3.2.2. Thermal Properties

**Figure 7:**
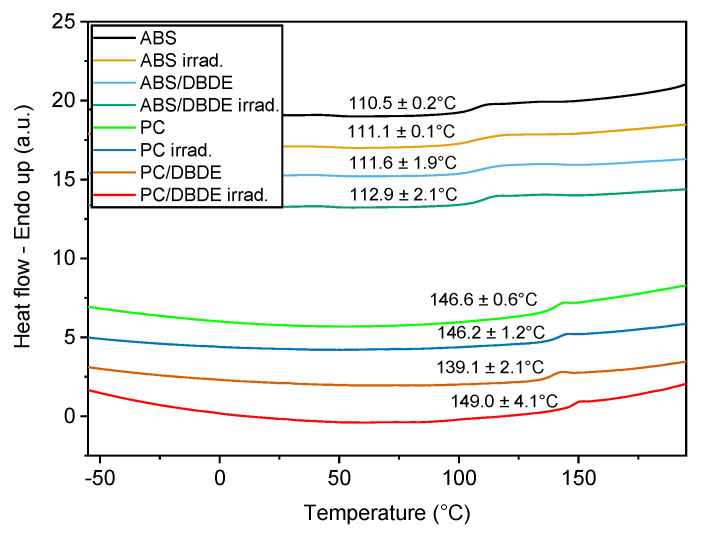
DSC thermograms of virgin and irradiated ABS, PC, 90 wt-% ABS/10 wt-% DBDE, and 90 wt-% PC/10 wt-% DBDE samples. The temperatures given in this figure correspond to the glass transition.

The DSC measurements were conducted on virgin and irradiated ABS, 90 wt-% ABS/10 wt-% DBDE, PC, and 90 wt-% PC/10 wt-% DBDE samples. As expected, a single glass transition temperature (Tg) was observed for each case, in the whole temperature range investigated (Figure 7). Interestingly, the addition of DBDE to the virgin polymer as well as the irradiation of these samples did not modify substantially the glass transition for a given polymer. Only the irradiated 90 wt-% PC/10 wt-% DBDE sample presented a considerable increase in the Tg as illustrated in Figure 7. Indeed, the averaged measured Tg of the latter sample exceeded the Tg of the virgin 90 wt-% PC/10 wt-% DBDE by 9 °C, which might be explained by the polymeric crosslinking effects [46].

On the other hand, the ABS containing samples exhibited only a low tendency for the crosslinking effects. An increase of 2 °C was observed for the irradiated 90 wt-% ABS/10 wt-% DBDE when compared to the virgin sample. This value was situated within the standard deviation obtained for the seven independent measurements. However, the Tg of the butadiene part of ABS, which might undergo a shift [47], was not detected in this study.

#### 3.2.3. Mechanical Properties

**Figure 8:**
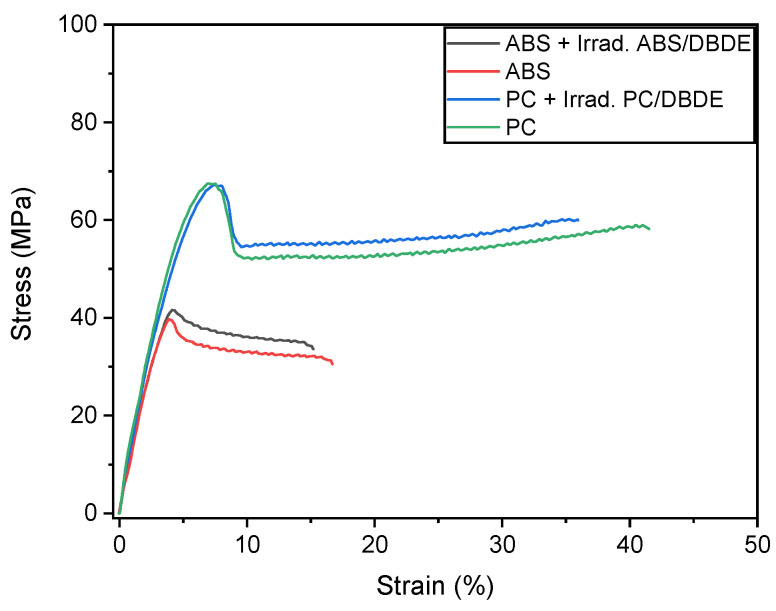
Stress-strain behavior of virgin ABS and PC and samples composed of 90 wt-% virgin ABS + 10 wt-% of the irradiated blend (90 wt-% ABS/10 wt-% DBDE), and 90 wt-% virgin PC + 10 wt-% of the irradiated blend (90 wt-% PC/10 wt-% DBDE).

The mechanical properties of the UV-visible light exposed polymers are expected to be significantly affected by the irradiation process [48]. Consequently, small quantities of the irradiated polymer/DBDE mixtures were incorporated into the virgin polymers in order to valorize these new decontaminated materials. Figure 8 presents a comparison of the averaged stress-strain curves of the virgin ABS and PC, as well as the following blends: “90 wt-% ABS + 10 wt-% of irradiated (90 wt-% ABS/10 wt-% DBDE)” and “90 wt-% PC + 10 wt-% of irradiated (90 wt-% PC/10 wt-% DBDE)”. Table 1 summarizes the data obtained from the static mechanical analysis.

Figure 8 shows that the stress-strain curves of the above-mentioned blends were not significantly affected by the presence of the irradiated part. The curves remain rather similar to those of the corresponding virgin polymers. Indeed, the incorporation of the 10 wt.-% irradiated material did not affect significantly neither elongation and strain at break nor strain at yield. Only a slight change in the Young moduli was noticed in the case of ABS. Table 1 also gathers the calculated data for the moduli of resilience and toughness. The modulus of resilience, which represents the strain energy per unit volume when the sample is being deformed up to its elastic limit, can be approximated as the area under the stress-strain curve up to the yield point. On the other hand, the modulus of toughness corresponds to the strain energy density that the sample absorbs before it fractures and was calculated as the total area under the stress-strain curve up to the fracture point. As shown in Table 1, only small differences were found for these moduli when comparing pristine with irradiated samples.

These results clearly demonstrate that the irradiated (90 wt-% ABS/10 wt-% DBDE) and (90 wt-% PC/10 wt-% DBDE) mixtures could be incorporated as a minor phase into virgin ABS and PC without affecting the mechanical properties of the virgin polymers. These findings confirm the possibility to valorize polymer/BFR waste by their exposure to UV-visible light.

#### 3.2.4. Molecular Weights and Polydispersities

**Figure 9:**
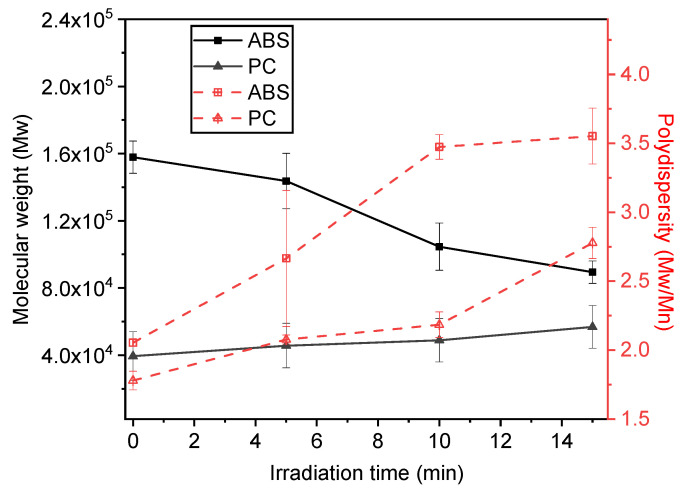
GPC results: Evolution of molecular weight and polydispersity of ABS and PC from 90 wt-% ABS/10 wt-% DBDE and 90 wt-% PC/10 wt-% DBDE films as function of irradiation time.

The effect of UV-visible irradiation on the evolution of the molecular weights was investigated as a function of the light exposure time for ABS and PC, recovered from virgin and irradiated 90 wt-% ABS/10 wt-% DBDE and 90 wt-% PC/10 wt-% DBDE films, respectively. Since the GPC analysis takes place in an organic solvent, the insoluble crosslinked compounds, eventually formed during photolysis, must be filtered off before the sample analysis. Thus, Figure 9 presents results from linear polymer chains, which were soluble in the organic solvent used for GPC measurements (THF).

An overall increase in the polydispersity, expressed by Mw/Mn, was observed for both polymers as shown in Figure 9, with a pronounced effect for ABS when compared to PC. Mw and Mn represent the weight and number averages of the molecular weight, respectively. The molecular weight of PC did not change significantly when increasing the exposure time to UV-visible light, indicating that PC presents a good stability towards the irradiation effects. On the other hand, ABS showed a strong decrease in the molecular weight when increasing the irradiation time; i.e., from Mw = 1.6 × 10^5^ g/mol for the virgin polymer to 9 × 10^4^ g/mol for the sample irradiated for 15 min. Such behavior has already been reported in the literature in relation to the chain scission effects of the irradiated polymers [36,41,48]. Going along these lines, it should be mentioned that ABS becomes increasingly insoluble in the THF with light exposure time, indicating the presence of both cross-linking and chain scission effects (see also Figure 6a). The total insoluble fraction corresponds roughly to 20% for ABS for an irradiation time of 15 min, whereas in the case of PC, the insoluble fraction is represented by under 5% for the same light exposure time.

## 4. Conclusions

In this study, the enhanced efficiency of a photodegradation process of DBDE under reduced pressure was observed when films composed of 90 wt-% ABS/10 wt-% DBDE and 90 wt-% PC/10 wt-% DBDE were irradiated by exposure to UV-visible light. Interestingly, the UV-visible light spectrum of the applied irradiation source was close to that of sunlight. Moreover, polymer properties of ABS and PC were mainly recovered after the irradiation process. This photodegradation technique could hence be a promising alternative to decontaminate brominated plastic waste for recycling purposes. Therefore, more research will be carried out to explore this possibility by studying the degradation of other BFRs as well as their generated photoproducts in various polymer matrices, and to investigate the faith and recovery prospects of the removed bromine compounds.

## Figures and Tables

**Table 1:** Summary of tensile properties of virgin ABS and PC, and samples composed of 90 wt-% virgin ABS + 10 wt-% of the irradiated blend (90 wt-% ABS/10 wt-% DBDE), and 90 wt-% virgin PC + 10 wt-% of the irradiated blend (90 wt-% PC/10 wt-% DBDE).

Sample	Young’s Modulus (MPa)	Stress at Yield (MPa)	Modulus of Resilience (J/m^3^)	Strain at Break (%)	Stress at Break (MPa)	Modulus of Toughness (J/m^3^)
Virgin ABS	1454 ± 70	40 ± 0.5	5 × 10^5^	18 ± 2	31 ± 2	5.3 × 10^6^
90 wt-% virgin ABS + 10 wt-% of the irradiated (90 wt-% ABS/10 wt-% DBDE) blend	1755 ± 45	43 ± 2	6.7 × 10^5^	16.8 ± 2.5	32.6 ± 1.3	5.23 × 10^6^
Virgin PC	1940 ± 50	68 ± 0.5	1.32 × 10^6^	44.7 ± 5.4	56.0 ± 4.5	2.2 × 10^7^
90 wt-% virgin PC + 10 wt-% of the irradiated (90 wt-% PC/10 wt-% DBDE) blend	1880 ± 35	67 ± 1	1.38 × 10^6^	32.7 ± 2.5	54.0 ± 4.4	2.0 × 10^7^

## Data Availability

The data presented in this study are available on request from the corresponding author.
